# Using the fragment molecular orbital method to investigate agonist–orexin-2 receptor interactions

**DOI:** 10.1042/BST20150250

**Published:** 2016-04-11

**Authors:** Alexander Heifetz, Matteo Aldeghi, Ewa I. Chudyk, Dmitri G. Fedorov, Mike J. Bodkin, Philip C. Biggin

**Affiliations:** * Evotec (UK) Ltd., 114 Innovation Drive, Milton Park, Abingdon, Oxfordshire OX14 4RZ, U.K.; † Department of Biochemistry, University of Oxford, South Parks Road, Oxford OX1 3QU, U.K.; ‡ NMRI, National Institute of Advanced Industrial Science and Technology (AIST), 1-1-1 Umezono, Tsukuba, Ibaraki 305-8568, Japan

**Keywords:** chemical interactions, computational, drugs, fragment molecular orbital (FMO), G-protein-coupled receptor (GPCR), modelling, receptor, structure-based drug-discovery (SBDD)

## Abstract

The understanding of binding interactions between any protein and a small molecule plays a key role in the rationalization of affinity and selectivity and is essential for an efficient structure-based drug discovery (SBDD) process. Clearly, to begin SBDD, a structure is needed, and although there has been fantastic progress in solving G-protein-coupled receptor (GPCR) crystal structures, the process remains quite slow and is not currently feasible for every GPCR or GPCR–ligand complex. This situation significantly limits the ability of X-ray crystallography to impact the drug discovery process for GPCR targets in ‘real-time’ and hence there is still a need for other practical and cost-efficient alternatives. We present here an approach that integrates our previously described hierarchical GPCR modelling protocol (HGMP) and the fragment molecular orbital (FMO) quantum mechanics (QM) method to explore the interactions and selectivity of the human orexin-2 receptor (OX_2_R) and its recently discovered nonpeptidic agonists. HGMP generates a 3D model of GPCR structures and its complexes with small molecules by applying a set of computational methods. FMO allows *ab initio* approaches to be applied to systems that conventional QM methods would find challenging. The key advantage of FMO is that it can reveal information on the individual contribution and chemical nature of each residue and water molecule to the ligand binding that normally would be difficult to detect without QM. We illustrate how the combination of both techniques provides a practical and efficient approach that can be used to analyse the existing structure–function relationships (SAR) and to drive forward SBDD in a real-world example for which there is no crystal structure of the complex available.

## Introduction

G-protein-coupled receptor (GPCR)–ligand interactions are fundamental to almost all processes occurring in living organisms, and as such it is perhaps unsurprising that they are the targets of about 40% of all prescribed drugs [1–3]. What is surprising is that these drugs only target approximately 50 of the 800 known GPCRs [4]. Thus there is huge potential in terms of the number of targets for new therapies to be designed against [5]. Further progress of drug discovery for GPCRs is highly dependent on the understanding of structure–function relationships (SAR) and the interactions between the receptor and the small molecule (drug candidate) [4,6–8]. Currently there are no experimental or computational tools available that can provide an accurate list of interactions between the receptor and the ligand [9] and their complex chemical nature [10].

The efficiency and cost-effectiveness of any drug-discovery process is highly dependent on the availability of structural data regarding the target receptor and on the reliability of the data mining tools [6–8]. The recent breakthroughs in structural biology of GPCRs have resulted in the solving of over 100 structures of GPCR–ligand complexes representing over 30 unique GPCRs [7,11]. Nevertheless, the interpretation of interactions observed in the atomic-resolution structure is usually performed by ‘visual inspection’ or alternatively with a simple molecular mechanics (MM) model that cannot explain the full complexity of the molecular interactions [10]. In many cases, the affinity and reasons why a particular ligand interacts the way it does with its receptor remain unclear.

Recently several notable reports have been published [10,12–14] that emphasized the pivotal role of a large number of ‘non-obvious’, hidden-from-eye interactions such as CH/π [15,16], halogen/π [17], cation/π [18] and non-classical H-bonds [19] in receptor–ligand binding that are not properly parameterized in currently available force fields (FF) [13]. This problem can be handled with quantum mechanics (QM) methods, which have always been considered a reliable approach for the exploration of receptor–ligand interactions [20,21]. However, in spite of their many advantages, traditional QM methods are generally not feasible for large biological systems, due to their high computational cost [22].

**Figure 1:**
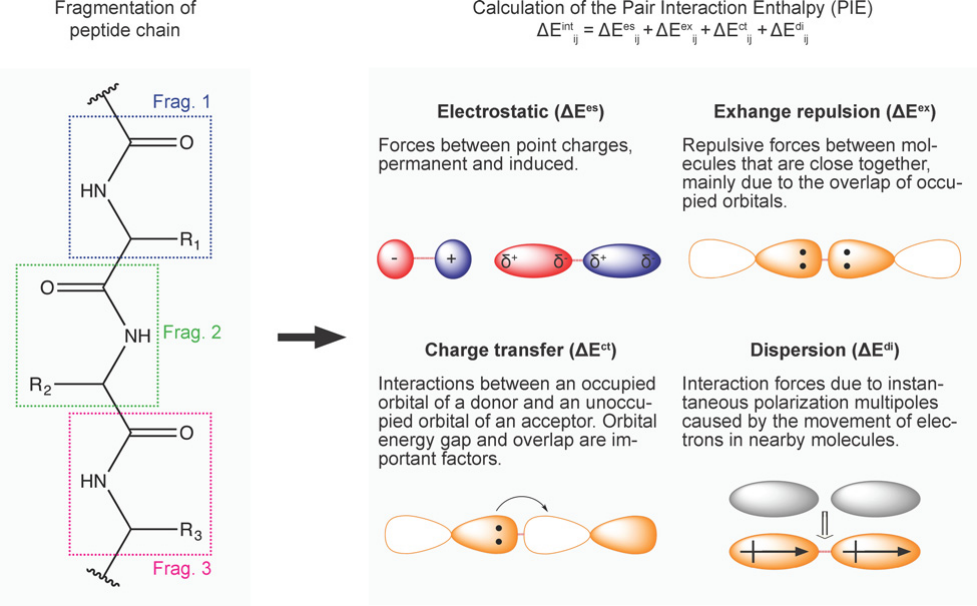
Workflow for PIEDA calculations and details on each of PIE terms that are computed [22] The electrostatic component arises from the Coulomb interaction between polarized charge distributions of fragments. The exchange repulsion term is derived from the interaction between fragments situated in close proximity and is always repulsive; it is due to the Pauli repulsion and is related to the overlap of two occupied orbitals. The charge transfer term arises from the interaction between occupied orbitals of a donor and unoccupied orbitals of an acceptor. The dispersion arises as the interaction between instantaneous dipole moments of two fragments. It is hydrophobic (non-polar) in nature and is obtained in PIEDA from the correlation energy of electrons.

The fragment molecular orbital (FMO) method [16,21,23] offers a considerable computational speed-up over traditional QM methods [24]. One of the key features of the FMO approach is that it can provide a list of the interactions formed between the ligand and the receptor and a chemically intuitive breakdown of these interactions [22]. Such information is essential for medicinal chemists to be able to rationally approach modification of lead compounds in order to increase favourable interactions. It works by dividing the system into smaller pieces called fragments (Figure 1). For example, in proteins, each residue can be represented as a fragment. Similarly, the ligand can be represented by single or multiple fragments as necessary. By performing QM calculations on fragments, one can achieve a high level of accuracy with very high efficiency.

The pair interaction energy (PIE) between any two fragments calculated by FMO is a sum of four energy terms: electrostatics, exchange-repulsion, charge transfer and dispersion, provided by pair interaction energy decomposition analysis (PIEDA) [25]–see Figure 1. The electrostatic and charge transfer terms are important in salt-bridges, hydrogen bonds and polar interactions, whereas the dispersion term can be thought of as hydrophobic in nature. The role of hydrophobic interactions is vital for biomolecular recognition but there is still no reliable predictive method for its quantification [10]. The exchange-repulsion term describes the steric repulsion between electrons [22] that prevents atoms from collapsing into each other.

The key difference between FMO and MM methods derives from the fact that FMO takes into account polarization and charge transfer [16,26]. The description of electrostatics in most FF is based on static charges that neglect polarization and in polar systems such as proteins they are an approximation to the actual state. The van-der-Waals forces, despite being generally well parameterized on average, are not capable of detecting the directional nature of the dispersion terms involving halogens [27]. Reported examples [28] comparing FMO and MM, have shown that the FMO method clearly outperformed FF-based scoring functions and demonstrated a high correlation with experimentally measured values of protein–ligand affinity [28,29]. In our recent report [29] we described how FMO has been applied for the analysis of 18 GPCR–ligand crystal structures representing different branches of the GPCR genome. This work revealed key and consensus interactions that are involved in receptor–ligand binding and were previously omitted from structure-based descriptions, including hydrophobic interactions, non-classical hydrogen bonds and the involvement of backbone atoms.

There is no need to compromise today in performing detailed analysis of protein–ligand structures using MM/FF whereas a similar analysis can be done with FMO that is reasonably quick. A typical FMO calculation on a ligand–receptor complex takes approximately 4 h on a 36 CPU cores to complete, significantly faster than weeks to a month (or more) for traditional QM approaches that have been used for estimating binding free energies.

Although X-ray crystallography is the preferred start point for structure-based drug-discovery (SBDD), in the context of a drug-discovery programme it is often the case that time prohibits its use for a series of complexes. The hierarchical GPCR modelling protocol (HGMP) has been developed to support GPCR SBDD programmes. HGMP has been successfully applied in GPCR drug discovery projects such as MCH-1R for obesity treatment [30], the orexin-1 and -2 receptors (OX_1_R and OX_2_R) for insomnia [31,32], the 5-HT_2C_ for the treatment of metabolic disorders [33,34] and in other confidential drug discovery programmes. Additionally, the HGMP technology was used in the solving of the two H_1_R crystals structures [4] bound to the second and third generation antihistamines: cetirizine and fexofenadine.

**Figure 2:**
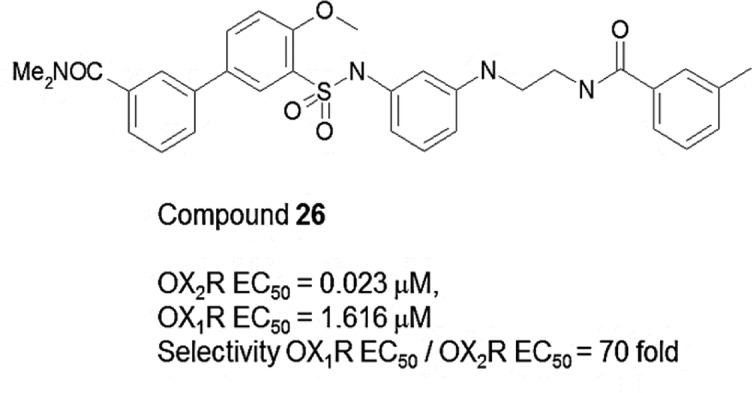
First nonpeptidic OX_2_R compound **26** discovered by Nagahara et al. [35].

In this work, we review how the integrated HGMP–FMO approach was applied to investigate the binding and selectivity of a recently reported nonpeptidic OX_2_R agonist (compound **26**, see Figure 2) and its closely related analogues [35]. We demonstrate that the FMO approach can be successfully applied to explore interactions in GPCR–ligand models rather than just crystallographic structures as previously reported [15,16,28,29,36,37].

## Hierarchical GPCR modelling protocol

The HGMP was developed by Evotec Ltd in conjunction with the University of Oxford to support SBDD programmes [30,31]. The HGMP generates a 3D model of GPCR structures and its complexes with small molecules by applying a set of computational methods. The models produced by HGMP are then used in drug discovery. The HGMP involves homology modelling followed by optimization protocols and flexible ensemble docking to predict binding poses and function of ligands bound to GPCRs. The HGMP includes a large set of integrated protocols like MD simulation [31], LowModeMD [30,38] and others to refine the GPCR models and exclusive scoring functions like the GPCR-likeness assessment score (GLAS) to evaluate model quality [31]. The HGMP has been successfully applied in a large number GPCR drug discovery projects and also to support crystallography.

## FMO technology

FMO is a general QM method that can be applied to any set of atoms, no matter if it is a soluble or membrane protein as described previously for exploration of the role of none-classical CH/π hydrogen bond in ligand recognition of β2-adrenergic GPCR receptor [15], for analysis of the GPCR–ligand crystal structures [29], for potency calculations on a novel Hsp90 fragment-linked inhibitor [39] and others. The FMO method can give a breakdown of the interaction energy between any two fragments and is consists of a sum of four pairwise interaction energy (PIE) terms: electrostatics, exchange-repulsion, charge transfer and dispersion (Figure 1). It is important to emphasize that the PIE is not a difference between energies of ‘free’ and ‘bound’ ligand but it rather represents the ‘strength’ of the interaction between the ligand and protein residues in the complex. Residues within a radius of ≤4.5 Å (1 Å=0.1 nm) around the ligand atoms often included in the FMO calculations. Based on the previous reports [29] we considered any interaction with an absolute PIE greater than or equal to 3.0 kcal/mol to be significant.

## Medical significance of nonpeptidic OXR agonists

Class A GPCRs; OX_1_R and OX_2_R, are located mostly in the brain and associated with a range of different physiological functions, including the control of feeding, energy metabolism, modulation of neuro-endocrine function and the regulation of the sleep−wake cycle. Two non-selective neuropeptides orexin-A (OxA) and orexin-B (OxB) are natural agonists of OX_1_R and OX_2_R, which have dual activity, at both receptors. The dual activity aspect of these peptides has limited the usefulness of these natural agonists as probe compounds to dissect out the precise role of each receptor, in several conditions related to OXR activation. The peptides are also inefficient for *in vivo* studies due to lack of ability to penetrate the blood–brain barrier (BBB). Small-molecule agonists of OXR are important for both research and medicine as having the potential to address both these problems of selectivity and BBB penetration. A wealth of data so far suggests that OXR agonists could be used for the treatment of sleep disorders, narcolepsy, cataplexy, obesity, hypophagia, as well as attention deficit hyperactivity, depression and related bipolar disorders [35,40–43]. Furthermore, it was demonstrated that OX_1_R agonists might be promising candidates for colon cancer therapy [44]. Activation of OX_1_R can drive apoptosis in human colon cancer cells and even reverse the development of established tumours.

However, in spite of their medical importance, the design of small-molecule agonists (rather than antagonists of peptide-activated GPCRs), is considered as one of the big challenges in drug discovery [31]. This is because for agonists, there is the added requirement that it must not only bind the receptor but also activate it. Peptide-activated GPCRs like OX_1_R and OX_2_R, are considered especially challenging due to the large number of specific and non-specific interactions that are usually involved in peptide binding and activation.

## FMO study of OX_2_R–agonist (compound **26**) complex

Through an extensive synthesis and screening programme, Nagahara et al. [35] recently reported the discovery of the first selective nonpeptidic OX_2_R agonists culminating in compound **26** (Figure 2). This new chemical screening information along with the recently solved OX_2_R crystal structure [45] (PDB entry 4S0V) provides a new opportunity to develop drugs against this important target. As we do not yet have a crystal structure for the OX_2_R in complex with compound **26**, the application of protocols such as the HGMP–FMO becomes the method of choice to advance the discovery of new ligands via the generation of plausible binding hypotheses that can be experimentally tested.

In previous site-directed mutagenesis (SDM) studies it was shown that alanine mutations of T111^2.61^, Q134^3.32^, D211^ECL2^, W214^ECL2^, Y223^5.38^, F227^5.42^, F346^7.35^ and H350^7.39^ caused a large (>50-fold) decrease in the potency of endogenous agonist without affecting the efficacy compared with WT. The mutations Y232A^5.47^ and Y317A^6.48^ resulted in a reduction of both EC_50_ (by 28.4- and 17.7-fold respectively) and *E*_max_ of 44.9% and 49.6%. These mutations caused a moderate decrease in potency of endogenous agonist (by 22.3-fold) without affecting its efficacy. These SDM data suggest that there is no clear correlation between the importance of residues for potency and for efficacy.

**Figure 3:**
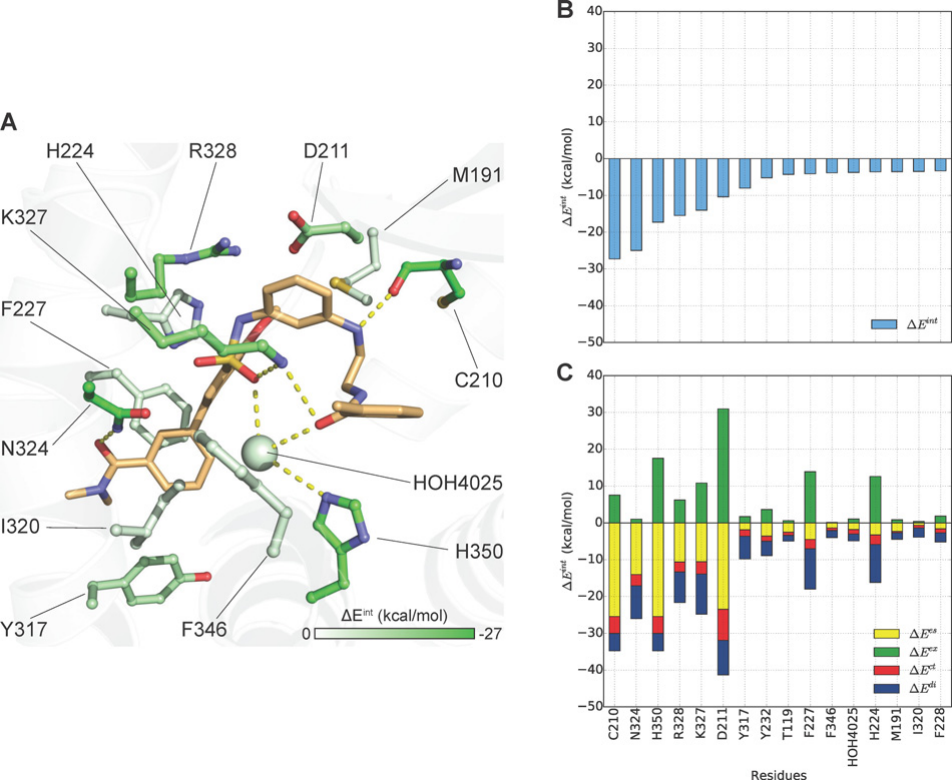
‘L’-shaped docking pose of compound 26 FMO results for (**A**) Literature-like ‘L’ shape docking pose as reported by Nagahara et al. [35]. The carbon atoms of the ligand are shown in light orange and for the receptor are coloured according to PIE values calculated by FMO. Nitrogen atoms are shown in blue, oxygen in red, sulfur in yellow and chlorine in light green. (**B**) Plot describes sorted PIE of the most significant residues and (**C**) plots describe the PIEDA of these key interactions. PIE terms: electrostatics, dispersion, charge-transfer and exchange repulsion are coloured coded yellow, blue, red and green respectively.

**Figure 4:**
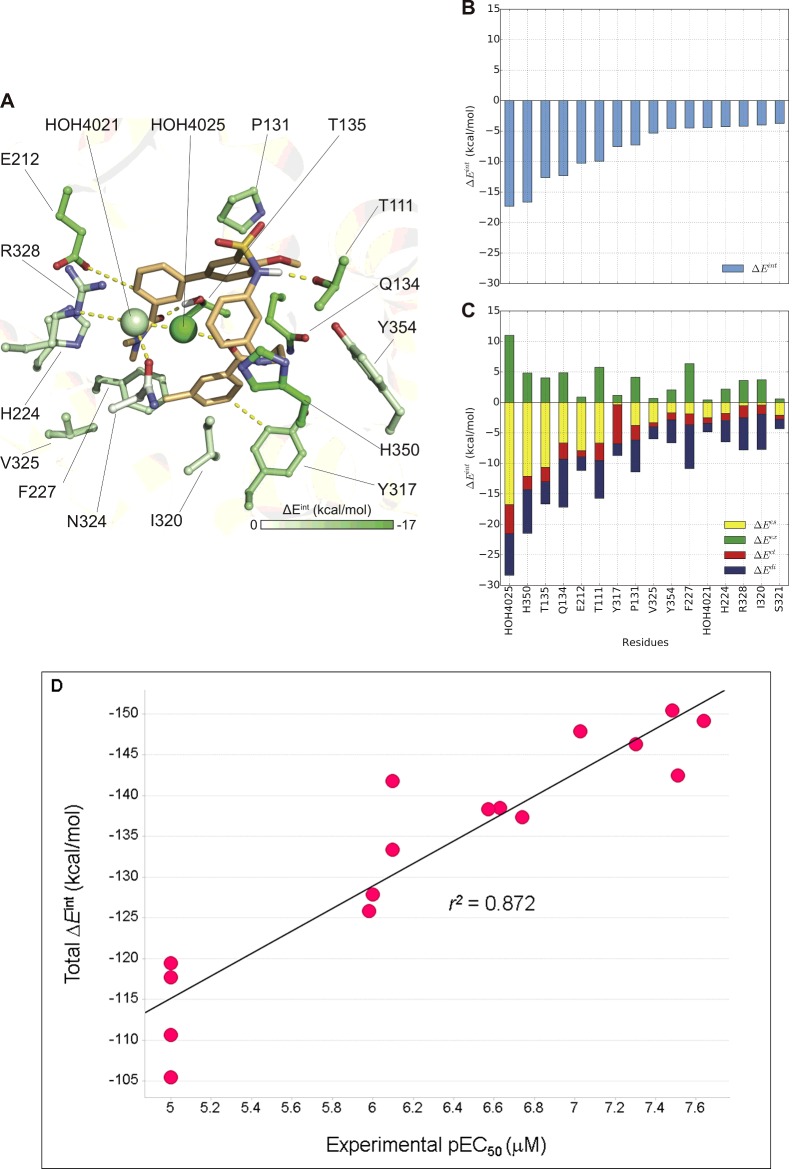
‘U’-shaped docking pose of compound 26 FMO results for (**A**) Suvorexant like ‘U’ shape docking pose. The carbon atoms of the ligand are shown in light orange and for the receptor are coloured according to PIE values calculated by FMO. Nitrogen atoms are shown in blue, oxygen in red, sulfur in yellow and chlorine in light green. (**B**) Plot describes sorted PIE of the most significant residues and (**C**) plots describe the PIEDA of these key interactions. PIE terms: electrostatics, dispersion, charge-transfer and exchange repulsion are coloured coded yellow, blue, red and green respectively. (**D**) Correlation plot between the experimentally measured pEC_50_s (EC_50_ data and structures of 16 analogues of compound **26** were taken from Tables 1 and 2 in Nagahara et al. [35]) and the total PIE values as calculated by FMO. The docking protocol used to dock the SAR ligands was as reported by us previously [29] where compound **26** was used as the docking template.

We recently proposed [46] two potential binding modes of compound **26** with OX_2_R produced by the HGMP: (1) ‘L’ shape docking pose (Figure 3) and (2) ‘U’ shape (Figure 4) as the antagonist Suvorexant adopts according to the recently solved crystal structure of the complex with OX_2_R (PDB entry 4S0V [45]).

In the first ‘L’ pose FMO detected 16 key interaction between compound **26** and OX_2_R residues: T119^2.69^, M191^4.64^, C210^ECL2^, D211^ECL2^, H224^5.39^, F227^5.42^, F228^5.43^, Y232^5.47^, Y317^6.48^, I320^6.51^, N324^6.55^, K327^6.58^, Arg328^6.59^, F346^7.35^, H350^7.39^ and with one water molecule HOH4025. Furthermore, these modelling observations are directly supported by the published SDM data [31,47]: the role of D211^ECL2^, F227^5.42^ F346^7.35^, H350^7.39^ and particularly of the residue Y232A^5.47^ in the potency and efficacy of OX_2_R endogenous agonist suggest their involvement in **26** agonist activity. However the relatively highly exchange repulsion term of D211^ECL2^ can be slightly artificial due to the fact that this residue is located on the loop which is more challenging to model. The exact role of F/Y^5.47^ (Y232^5.47^ in OX_2_R) in class A GPCRs activation is not clear but it is frequently engaged in interaction with agonists [48].

In the ‘U’ shape (Figure 4) pose, FMO detected 13 residues: T111^2.61^, P131^3.29^, Q134^3.32^, T135^3.33^, E212^ECL2^, H224^5.39^, F227^5.42^, Y317^6.48^, I320, V325^6.56^, R328, H350^7.39^, Y354^7.43^ and two water molecules: HOH4021 and HOH4025, that are involved in **26** binding. This pose is also supported by SDM data with residues T111^2.61^, Q134^3.32^, F227^5.42^ and H350^7.39^ and particular the interactions with Y317^6.48^. The interactions with the toggle switch residue Y317^6.48^ and with the aromatic cluster residue F227^5.42^, support the GPCR activation switch mechanism that allows compound **26** to have OX_2_R agonist function. This agonist-bound switch was proposed to be part of a larger ‘transmission switch’ that accounts for the relocation of conserved residues W^6.48^ (Y317^6.48^ in OX_2_R) and F^6.44^ towards P^5.50^ [48]. A potential explanation for OX_1_R selectivity arises from potential interactions with the non-conserved residues T111^2.61^ (S102^2.61^ in OX_1_R) and T135^3.33^ (A135^3.33^ in OX_1_R).

The total PIE energy in docking pose ‘L’ is −110.17 kcal/mol and in ‘U’ is −143.18 kcal/mol. This suggests that in binding mode ‘U’, compound **26** forms a more stable complex with OX_2_R. In both docking poses the relative contributions of electrostatic and hydrophobic (dispersion) interactions are equal. However, we should add a note of caution here in that these are docked poses of agonists based on an antagonist-bound crystal structure template. There is a possibility that the agonist-bound form of the receptor is one that is quite different from the antagonist-bound form.

Another interesting aspect that we evaluated here is the correlation between experimentally measured EC_50_s and PIE as calculated by FMO for analogues of compound **26** as published by Nagahara et al. [35]. It is known that ligand receptor affinity and efficacy might be driven by different energy terms including direct enthalpic contributions, entropy, solvation and the ‘strain energy’ of the ligand's bioactive conformation [10]. Despite the fact that not all these factors are accounted for we observed significant correlation (*r*^2^=0.872, Figure 4D) between experimental values of EC_50_, measured for 16 analogues of compound **26** and the PIE calculated for their ‘U’ poses (no significant correlation was observed for the ‘L’ pose). These results are in agreement with our previously published report [29] where we demonstrated significant correlation between PIEs and experimentally measured affinities of OX_2_R Suvorexant-based antagonists. These observations increase our confidence that FMO can be used to provide additional insight into SBDD against GPCR targets even for modelled GPCR–ligand complexes.

FMO can be a highly useful tool for rational SBDD [16,39,49], as it provides an accurate and comprehensive list of strong, weak or repulsive interactions between the ligand and its surrounding residues. Such information is highly useful to guide modifications, substitution, scaffold hoping, linking or extension of chemical moieties to form stronger or new interactions with the protein or alternatively to remove repulsions. FMO can also be helpful in analysis of the ligand–water–protein network, to distinguish between energetically favourable and unfavourable water molecules and to design ligands that can interact or displace certain waters. As previously demonstrated [29], significant correlation between protein–ligand affinity and FMO energy terms [28] indicates that they can be efficiently used as descriptors in QSAR modelling to predict the binding affinities of new molecules. FMO has been successfully applied in the discovery of a novel Hsp90 inhibitors [39] and in many other our confidential drug discovery programmes. We learned that application of the FMO in hit-to-lead and lead optimization stages of drug discovery is a highly efficient for the design, evaluation and filtering of targets for synthesis that significantly decreases the effort, time and cost of chemical synthesis.

## Conclusions

Here we present a new approach that opens an alternative avenue for the structural exploration of GPCRs and structure-based GPCR drug discovery. We have applied this approach to explore the binding and selectivity of an OX_2_R agonist. The outcome of this study is currently being applied in the generation of new OX_2_R agonists and for the discovery of first in class OX_1_R agonists. To our knowledge this is the first time that FMO calculations have been applied to docking poses of ligands in a GPCR. Applying the FMO analysis to this kind of problem results in two distinct benefits: (a) complex QM theories are condensed into four simple and intuitive quantities and (b) calculations become much faster than traditional QM approaches. This knowledge can be used to understand the chemical nature of existing receptor–ligand complexes, make suggestions for mutations and more importantly can suggest rational ligand optimization routes. The HGMP–FMO approach creates a cost-efficient new avenue for a SBDD against GPCR targets.

## Abbreviations

BBB: blood–brain barrier
FF: force field
FMO: fragment molecular orbital method
GPCR: G-protein-coupled receptor
HGMP: hierarchical GPCR modelling protocol
MM: molecular mechanics
OX_1_R: orexin-1 receptor
OX_2_R: orexin-2 receptor
PIE: pair interaction energy
PIEDA: pair interaction energy decomposition analysis
QM: quantum mechanics
SAR: structure–function relationships
SBDD: structure-based drug discovery
SDM: site-directed mutagenesis
